# Itemization difference of patient-reported outcome in patients with chronic liver disease

**DOI:** 10.1371/journal.pone.0264348

**Published:** 2022-02-22

**Authors:** Ming-Chieh Lin, Chia-Yen Dai, Chung-Feng Huang, Ming-Lun Yeh, Yi-Chan Liu, Po-Yao Hsu, Yu-Ju Wei, Pei-Lun Lee, Ching-I Huang, Po-Cheng Liang, Ming-Yen Hsieh, Meng-Hsuan Hsieh, Tyng-Yuan Jang, Zu-Yau Lin, Jee-Fu Huang, Ming-Lung Yu, Wan-Long Chuang

**Affiliations:** 1 Hepatobiliary Division, Department of Internal Medicine, Kaohsiung Medical University Hospital, Kaohsiung, Taiwan; 2 Graduate Institute of Clinical Medicine, Kaohsiung Medical University, Kaohsiung, Taiwan; 3 Faculty of Internal Medicine, College of Medicine, Kaohsiung Medical University, Kaohsiung, Taiwan; 4 Department of Internal Medicine, Kaohsiung Municipal Ta-Tung Hospital, Kaohsiung Medical University, Kaohsiung, Taiwan; 5 Department of Internal Medicine, Chi-Mei Medical Center Liou-Ying, Tainan, Taiwan; Universitas Indonesia Fakultas Kedokteran, INDONESIA

## Abstract

**Background and aims:**

The itemization difference of patient-reported outcome (PRO) in hepatitis patients with different etiologies remains elusive in Asia. We aimed to assess the characteristics and the difference of health-related quality of life (HRQoL) in chronic hepatitis B (CHB), chronic hepatitis C (CHC), and non-alcoholic fatty liver disease (NAFLD) patients.

**Methods:**

We conducted the study in an outpatient setting. The 36-Item Short Form Health Survey (SF-36) was completed by the patients upon the initial diagnosis and recruitment for a long-term follow-up purpose. The PRO results were also assessed by disease severity.

**Results:**

There were 244 patients (198 males) of CHB, 54 patients (29 males) of CHC, and 129 patients (85 males) of NAFLD, respectively. CHC patient had the mean score of 67.1 ± 23.3 in physical component summary (PCS) of the SF-36 health survey, which was significantly lower than CHB patients (76.4 ± 19.5), and NAFLD patients (77.5 ± 13.7), respectively (p = 0.001). The significantly lower performance of PCS in CHC patients was mainly attributed to the lower performance in physical functioning and bodily pain components. Higher fibrosis 4 index scores were significantly associated with lower PCS scores in all patient groups. There was no significant difference of mean mental component summary (MCS) between groups. However, NAFLD patients had significantly lower mental health scores than other groups (p = 0.02).

**Conclusions:**

The significant difference of HRQoL exists in hepatitis patients with different etiologies. Disease severity leads to a lower PCS performance.

## Introduction

Hepatitis is a worldwide huge health concern, largely due to its subsequent sequela such as cirrhosis, liver failure and hepatocellular carcinoma (HCC). The causes and/or etiologies vary in different regions globally. Viral hepatitis infections, mainly hepatitis B virus (HBV) and C virus (HCV), are prevalent in Asia-Pacific region. Fortunately, the rapid progress of therapeutic and diagnostic strategies in viral hepatitis infections has largely changed the landscape of management [1]. However, the accumulated health burden of viral hepatitis infections has been significantly exacerbated by a global increase of non-alcoholic fatty liver disease (NAFLD) in parallel to the global westernization [1–4]. The natural course and disease mechanisms in NAFLD as well as the optimal lifestyle intervention and therapeutic potentials remain elucidation.

On the other side, the extrahepatic manifestations are not uncommon in patients with viral hepatitis infections. For example, chronic HCV infection (CHC) may lead to type 2 diabetes mellitus (T2DM), hyperlipidemia, cryoglobulinemia, lymphoma, atherosclerosis, ischemic heart, glomerulonephritis and autoimmune disorders [5–8]. Meanwhile, NAFLD generally is the hepatic manifestation of metabolic abnormalities such as T2DM, hyperlipidemia, hypertension, and ischemic heart disease. Therefore, the affected may not only suffered from the liver injuries but also from the insults of the extrahepatic disorders [9]. Therefore, the comprehensive assessment based on the patient-centered approach is the essential step both for disease severity clarification and for long-term follow-up.

In the initial approach for the newly-diagnosed patients, patient reported outcome (PRO) is an easily overlooked item in a clinical setting. In the many directions of PRO, health-related quality of life (HRQoL) is an important field for elucidation. Besides mental and social concerns, the associated disorders may also have an impact on HRQoL of the patients. Evaluating the difference of HRQoL in these patients could give us not only a patient-oriented perspective of the disease, but also an indicator for assessing the patient’s perception in terms of disease awareness and medical adherence.

Evaluating the HRQoL in hepatitis patients is a clinical challenge largely due to the asymptomatic presentation of viral hepatitis infections and NAFLD. Meanwhile, the mental health of those affected patients is intentionally or unintentionally underestimated in Asia-Pacific, partly due to traditional culture burden. Taiwan has a high prevalence of both viral hepatitis infections and NAFLD. The estimated prevalence of NAFLD was >40% in Taiwanese community-based studies, whilst the prevalence of chronic hepatitis B (CHB) and CHC reached 12–15%, and 4–9%, respectively [1, 10–12]. Therefore, the unique background thus provides an excellent field to assess the difference of HRQoL in patients with different etiologies [13].

Consequently, we conducted the current study aiming to elucidate the HRQoL in chronic liver disease (CLD) patients. The characteristics and the difference of CLD of different etiologies were also be assessed.

## Materials and methods

The ethical committee of the Kaohsiung Medical University Hospital approved this study before it was performed (KMUHIRB-E1-20170279). This prospective cohort study was conducted at a medical center hospital, three regional hospitals, and a primary care center in southern Taiwan. During the period from January 2008 to August 2018. Those treatment-naïve patients who have been diagnosed of CHB, CHC or NAFLD for more than 6 months were consecutively enrolled. In this study, all patients received standard of care according to the regional management guidelines. We excluded patients who presented with a significant alcohol consumption history, defined as > 20 g ethanol/day in males and > 10 g ethanol/day in females. We also excluded patients who presented with autoimmune liver diseases, such as primary biliary cirrhosis, primary sclerosing cholangitis, haemochromatosis or Wilson’s disease. Written informed consent for an interview, anthropomorphic measurements, and medical record review were obtained from subjects prior to enrollment. Age, gender, and major comorbidities were recorded using standardized techniques.

We used Health Survey (SF-36) as a tool to survey HRQoL upon their outpatient-based visits. The SF-36 Health Survey is a 36-item, patient-reported survey of patient health. The SF-36 is a measure of health status and has been widely used globally with confident performance. The SF-36 consists of eight scaled scores, which are the weighted sums of the questions in their section. Each scale is directly transformed into a 0–100 scale on the assumption that each question carries equal weight. The lower the score shows the more disability. The eight sections consist physical component summary (PCS) and mental component summary (MCS). Physical functioning (PF), role limitation due to physical health (RP), bodily pain (BP) and general health (GH) constitute PCS, whereas vitality (VT), social functioning (SF), role limitations due to emotional health (RE), and mental health (MH) contribute to MCS. The patients were divided into three groups by disease diagnosis. Associated underlying systemic diseases were also documented form previous medical records, including type 2 diabetes mellitus, hypertension, hyperlipidemia, liver cirrhosis, and neuro-psychiatric disorders (anxiety, depression, insomnia or other diagnosed psychiatric diseases).

Liver disease severity were assessed in the all patients by using fibrosis 4 index (FIB-4), in which a higher score had a higher specificity and positive predictive value for fibrosis stage [14].

### Statistical analysis

The group means (standard deviation, SD) were compared using the independent sample t-test or one-way ANOVA. We used the Chi-square test or Fisher’s exact test to compare categorical variables of different groups. All statistical analyses were based on two-sided hypothesis tests with a significance level of p < 0.05. Quality control procedures, database processing and analyses were performed using the SPSS 12.0 statistical package (SPSS, Inc., Chicago, IL, USA).

## Results

A total of 452 CLD patients (312 males, mean age = 47.6 ± 13.2 years) diagnosed of CHB, CHC or NAFLD were consecutively enrolled. There were 244 patients (198 males, age = 44.2 ±11.2 years) of CHB, 54 patients (29 males, age = 55.2 ± 12.7 years) of CHC, and 129 patients (85 males, age = 50.9 ± 14.8 years) of NAFLD, respectively. The baseline characteristics were shown in Table 1.

**Table 1:** Demographic characteristics.

	CHB group	CHC group	NAFLD group
	*n = 244*	*n = 54*	*n = 129*
Age, years (mean ± SD)	44.2 ±11.2	55.2 ± 12.7	50.9 ± 14.8
Male, n (%)	198 (81.1%)	29 (53.7%)	85 (65.9%)
Type 2 DM, n (%)	39 (16.0%)	13 (24.1%)	42 (32.6%)
Hypertension, n (%)	48 (19.7%)	18 (33.3%)	61 (47.3%)
Hyperlipidemia, n (%)	77 (31.6%)	17 (31.5%)	89 (69.0%)
Liver cirrhosis, n (%)	42 (17.2%)	8 (14.8%)	4 (3.1%)
^†^Neuro-psychiatric disorders, n (%)	24 (9.8%)	24 (44.4%)	75 (58.1%)

CHB: chronic hepatitis B patient; CHC: chronic hepatitis C patient; NAFLD: non-alcoholic fatty liver disease patient; SD: standard deviation, DM: diabetes mellitus.

^†^Neuro-psychiatric disorders: anxiety, depression or insomnia.

### HRQoL in different groups

In the physical components of the SF-36 health survey, CHC patients had significant lower performance of PCS (mean = 67.1 ± 23.3) scores than CHB and NAFLD patient groups (p = 0.001), particularly in the items of PF (mean = 81.6 ± 16.3) and BP (mean = 75.7 ± 23.0) (Table 2).

**Table 2:** Comparison of SF-36 scores between CHB, CHC and NAFLD patient groups.

	CHB group	CHC group	NAFLD group	p value
n	244	54	129	
***PCS***	***76*.*4 ± 19*.*5***	***67*.*1 ± 23*.*3***	***77*.*5 ± 13*.*7***	***0*.*001***
PF	89.8 ± 15.2	81.6 ± 20.6	88.2 ± 16.3	0.001
RP	75.1 ± 35.9	67.4 ± 41.9	76.6 ± 22.5	0.166
BP	83.4 ± 20.9	75.7 ± 23.0	88.1 ± 16.0	<0.001
GH	54.1 ± 19.1	54.5 ± 19.9	53.5 ± 14.2	0.925
***MCS***	***62*.*0 ± 22*.*7***	***67*.*8 ± 25*.*4***	***61*.*7 ± 21*.*3***	***0*.*116***
VT	59.5 ± 17.9	61.6 ± 21.0	57.6 ± 14.8	0.292
SF	81.1 ± 18.8	81.8 ± 20.5	83.7 ± 14.0	0.384
RE	76.7 ± 36.7	74.0 ± 38.6	78.3 ± 22.1	0.695
MH	65.1 ± 17.0	70.3 ± 21.4	63.2 ± 13.8	0.019

CHB: chronic hepatitis B, CHC: hepatitis C, NAFLD: non-alcoholic fatty liver disease; PCS: physical component summary; PF: physical functioning, RP: role limitation due to physical health; BP: bodily pain; GH: general health; MCS: mental component summary; VT: vitality; SF: social functioning; RE: role limitations due to emotional health; MH: mental health.

**Fig 1:**
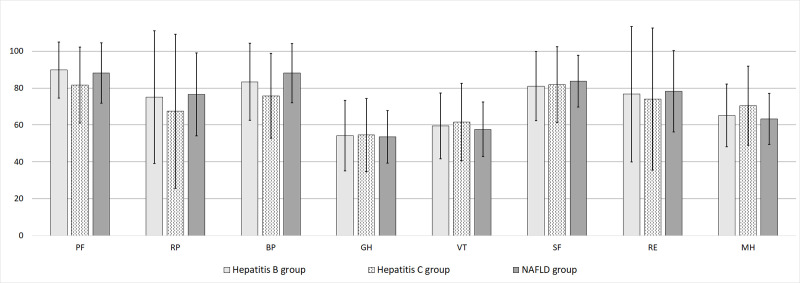
The eight items of SF-36 questionnaire in three patient groups (mean ± SD).

**Fig 2:**
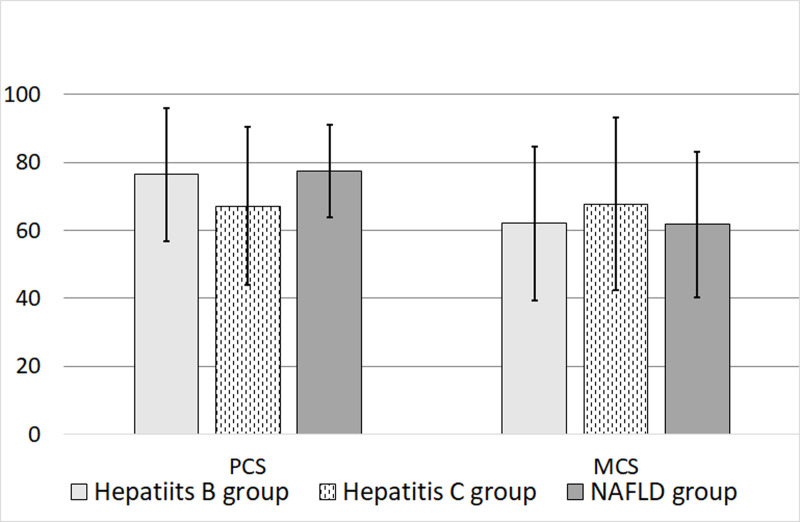
Respective PCS (physical component summary) and MCS (mental component summary) of the three patient groups (mean ± SD).

There was no significant difference between CHB and NAFLD patient groups in the PCS scores. In the MCS of the SF-36 survey, NAFLD patient groups had a significant lower MH score (mean = 63.2 ± 13.8) than CHB and CHC patient groups (p = 0.019) (Table 2). There was no significant difference in MCS scores between 3 groups. Comparison of mean scores of eight realms between groups are demonstrated as error bars in Fig 1, and respective summary scores in Fig 2.

### The correlation between disease severity and HRQoL

The FIB-4 index scores in CHB and CHC were 2.76 ± 3.44, and 2.89 ± 2.98, respectively. The scores of CHB and CHC patient groups were significantly higher than NAFLD patients (1.51 ± 1.09) (p< 0.001).

**Fig 3:**
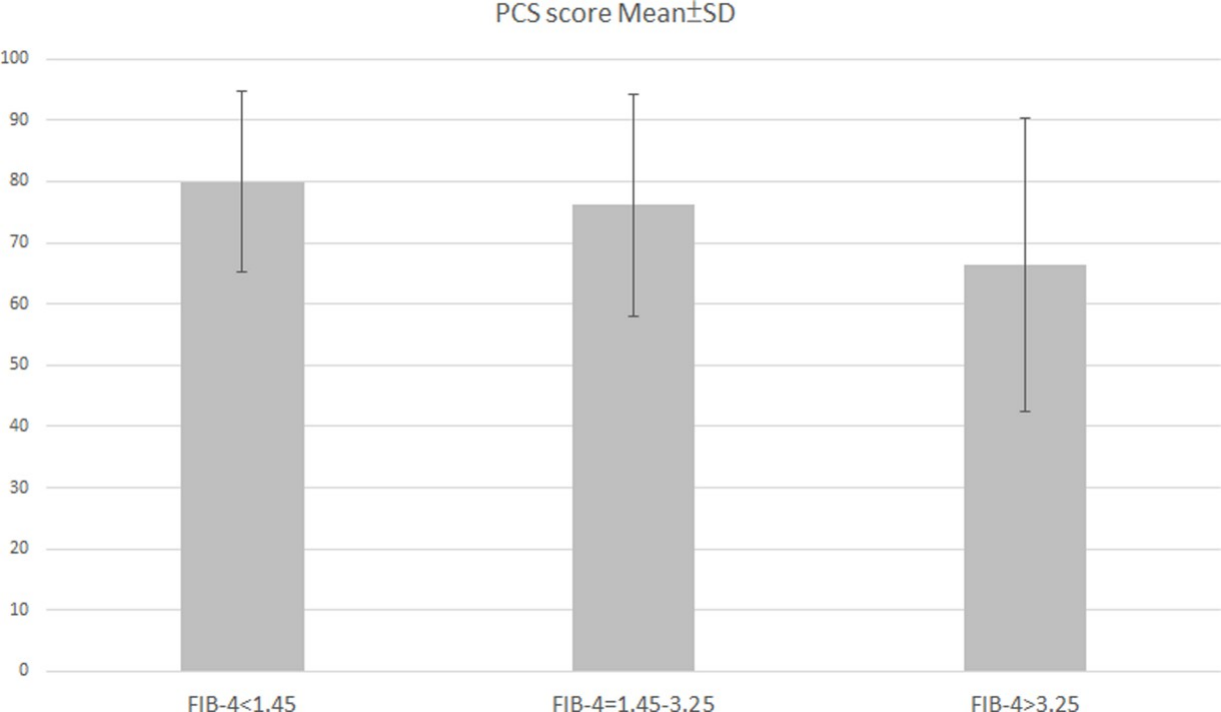
The correlation between PCS scores and FIB-4 scores.

Patients were divided into three groups by FIB-4 index scores, respectively FIB-4 score < 1.45, FIB-4 score between 1.45 and 3.25, and FIB-4 score > 3.25. Mean scores were 80.0 ± 14.7 in patients with FIB-4 scores < 1.45; 76.2 ± 18.2 in patients with FIB-4 scores between 1.45 and 3.25; and 66.3 ± 23.9 in patient with FIB-4 scores > 1.45 (Fig 3). Pearson correlation was calculated for linear association between FIB-4 index and SF-36 items. There was a significant inverse correlation between PCS scores and FIB-4 index (p< 0.001). By contrast, there was no significant correlation between MCS and FIB-4 index.

## Discussion

HRQoL remains a major concern of general health. A comprehensive understanding of HRQoL among CLD patients would give insight to physical and mental challenges the patients may face [15, 16]. The current study demonstrated the significant difference of HRQoL existed between CLD of different etiologies. Our itemization results showed that CHC patients had a lower PCS score, particularly in the items of BP and PF. PCS performance had an inverse correlation with disease severity in all patient groups. Although there was no significant difference of MCS between groups, we found that NAFLD patients had a significant lower MH performance compared to CHB and CHC patients. Our results thus provided the evidence for CLD patient care in a PRO setting.

CHB is an infectious disease prevalent in Asia-Pacific. A large proportion of the patients were infected during their early life. Therefore, the complex scenario of disease manifestation is mainly affected by the host-virus interaction during the long-term course. On the other side, CHC is a lymphotropic virus. There has been robust evidence showing there are various extrahepatic manifestations in CHC patients [10]. Previous studies demonstrated that CHC have negative impact on both physical and mental aspects of HRQoL [5, 17–19]. CHC involves changes from emotional, and social aspects that have a significant impact on HRQoL of CHC patients. Our results demonstrated that CHC patients had a lowest score in physical components. The subtle and various extrahepatic manifestations of CHC may largely contributed to the difference. Mover, the lymphotropic characteristic of HCV per se may lead to the susceptible development of neuropsychiatric abnormalities [5, 18–21]. Our results may also pave the way for a more sophisticated investigation of the pathogenic mechanisms of the mental health issues in the future.

Previous studies showing that a decreased HRQoL may exist and may vary in patients with different etiologies of hepatitis. Previous study by Dan et al. showed that CHB patients had the better results of HRQoL compared to CHC and NAFLD patients by using 29-item Chronic Liver Disease Questionnaire (CLDQ) [22]. Their results indicated that NAFLD patients had significantly lower quality of life scores compared with CHB or CHC on multiple CLDQ domains, suggesting that HRQL was severely impaired in patients with NAFLD. Previous studies demonstrated that NAFLD patients had a decreased HRQoL score, mainly in the domains of inability to perform daily tasks, fatigue, autonomic dysfunction and obesity [22–24]. The decreased performance might be due to the comorbidities such as cardiovascular disease, metabolic syndrome, obesity or malignancies [25, 26]. Our study echoed their results showing that NAFLD patients had a significant lower MH performance compared to CHB and CHC patients. Our study further stratified the HRQL items into PCS and MCS, which was a feasible approach in a patient-centered setting. Our results demonstrated that CHC patients had a lower PCS score, particularly in the items of BP and PF. On the other side, the MCS scores of the 3 groups were comparable. The discordant results may be attributed to the difference in terms of ethnicity, disease severity between groups, and assessment tools. Of note was that our patients were prospectively recruited from outpatient clinic and their CLD were newly-diagnosed for 6 months before their entry into a long-term follow-up study. The study design much decreased the confounding from at least the time factor. s Future studies will be needed for comparison of their PRO between different time points after clinical interventions.

Fibrosis is the major determining factor associated with outcomes of CLD [27]. The study demonstrated that disease severity, assessed in the all patients by using FIB-4, had an impact on the PRO. The higher FIB-4 scores were significantly associated with the lower PCS in all patient groups. By contrast, there was no significant correlation between MCS and FIB-4 scores. The difference of the impacts between PCS and MCS deserves further investigation. On the other side, our results demonstrated that the FIB-4 scores of CHB and CHC patient groups were significantly higher than NAFLD patients. The study was of a prospective, cross-sectional design in which the treatment-naïve patients were consecutively recruited upon their initial diagnosis. Therefore, the impact of disease severity across different etiology could be elucidated by a longitudinal observational study. Meanwhile, more precise disease severity assessment tools such as paired liver biopsies or stiffness elastography might be informative for clarifying the important issue.

There are some limitations in this research. Firstly, there was no control group of general population included in this study for comparison. The difference between CLD and normal population in the aspects of HRQoL and/or PRO deserves further exploration. Secondly, HRQoL might change significantly upon the initiation or success of antiviral therapies. Our results did not include those who received antiviral therapies for post treatment comparison in a longitudinal manner. The changes of HRQoL may vary between CHB and CHC after therapeutic interventions. Thirdly, the assessment tool depended on single method, and the scientific value could be strengthened by the validation of other questionnaires.

In conclusion, CLD patients of different etiologies presented with different performance of HRQoL items. CHC patients had decreased PCS, particularly in physical functioning and bodily pain, whereas NAFLD patients showed decreased MH in MCS domain. Disease severity had a significantly inverse correlation with PCS of HRQoL.
